# An Integrated Framework Coupling Hierarchical Hyperspectral *i*‐trait Screening, Gene Identification, and Functional Validation for Genetic Dissection of Salt Tolerance in Cotton

**DOI:** 10.1002/advs.78504

**Published:** 2026-10-26

**Authors:** Bowei Xu, Le Liu, Mengli Yu, Yuling Guo, Junli Ye, Jun Zhang, Rumeng Zhao, Jiajie Yang, Wanneng Yang, Yameng Xu, Liqiang Fan, Zuoren Yang

**Affiliations:** ^1^ State Key Laboratory of Cotton Bio‐Breeding and Integrated Utilization Institute of Cotton Research Chinese Academy of Agricultural Sciences Anyang Henan China; ^2^ Xinjiang Key Laboratory of Crop Gene Editing and Germplasm Innovation/Northwest Center of the National Technology Innovation Center for Saline‐Alkali Agriculture Institute of Western Agriculture of CAAS Changji Xinjiang China; ^3^ Henan Sesame Research Center Henan Academy of Agricultural Sciences Zhengzhou Henan China; ^4^ Wuhan Greenpheno Science and Technology Co., Ltd. Wuhan China; ^5^ College of Plant Science & Technology Huazhong Agricultural University Wuhan China

**Keywords:** cotton, GWAS, HSI, i‐traits, salt stress

## Abstract

Cotton is an economically pivotal fiber crop worldwide, but its growth and yield are critically limited by soil salinity. Salt tolerance is a complex quantitative trait, and conventional destructive phenotyping methods have long hindered the efficiency and accuracy of gene discovery and genetic dissection. To overcome this phenotyping bottleneck and establish a streamlined workflow from phenotype to gene, we developed an integrated framework coupling dynamic hyperspectral phenomics, hierarchical trait selection, genome‐wide association study (GWAS), and functional validation, and applied it to dissect the genetic basis of salt tolerance using a natural population of 419 upland cotton accessions. We first confirmed abundant salt tolerance phenotypic diversity in the population via four core physiological traits and their phenotypic plasticity indices. Using time‐series hyperspectral imaging (HSI), we extracted large‐scale image‐derived traits (*i*‐traits) capturing dynamic physiological responses to salt stress. A three‐tiered screening strategy integrating t‐test, broad‐sense heritability (H²) estimation, and multi‐algorithm machine learning consensus was applied to identify core salt‐responsive *i*‐traits with biological relevance, genetic stability, and low redundancy. Using these core *i*‐traits as phenotypic inputs for GWAS, we successfully mapped GhST7 as a key candidate gene. Functional validation via CRISPR/Cas9‐mediated knockout and haplotype‐specific overexpression demonstrated that *GhST7* acts as a key regulator of cotton salt stress responses, and natural variation in its promoter region confers differential salt tolerance by modulating gene expression levels. Collectively, this study not only identifies a functionally verified salt tolerance gene with breeding application value, but also establishes a coherent phenomics–genomics–functional validation framework, providing an efficient paradigm for high‐precision genetic dissection of complex stress‐tolerance traits in crops.

## Graphical abstract

An integrated phenomics–genomics framework captures dynamic salt‐stress responses in laboratory‐grown cotton seedlings through time‐series hyperspectral imaging and non‐destructive i‐trait extraction. Hierarchical screening identifies biologically relevant, heritable, and non‐redundant traits for high‐resolution GWAS, identifying GhST7. Subsequent functional analyses show that promoter haplotypes regulate GhST7 expression, antioxidant capacity, and cotton salt tolerance.

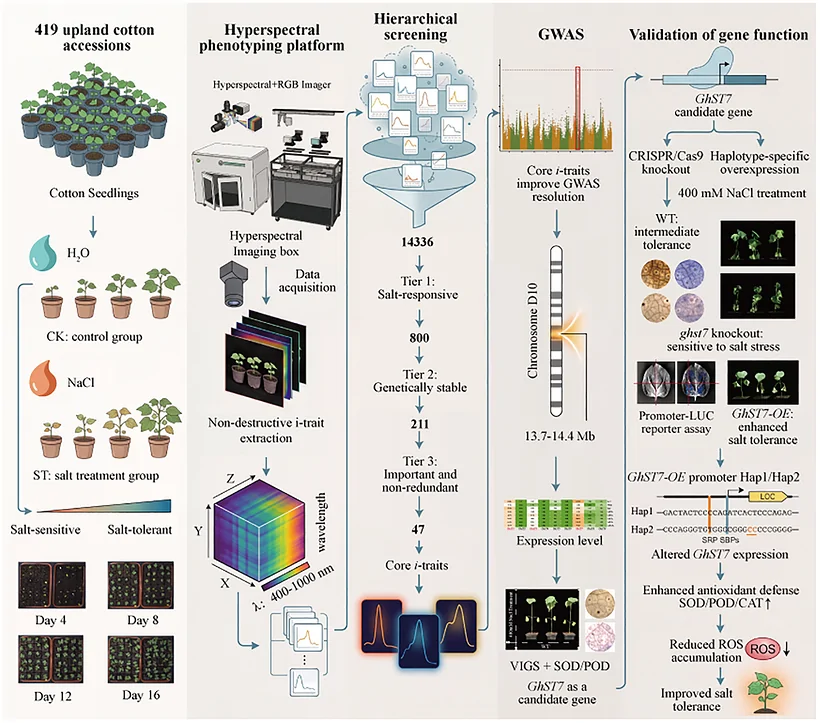

## 1 Introduction

Soil salinity stress ranks among the most widespread and deleterious abiotic stressors, severely restricting global agricultural productivity [1, 2]. Excessive soluble salts in the rhizosphere trigger ionic toxicity, hyperosmotic stress, and secondary oxidative damage. These stresses disrupt nutrient and water uptake, impair photosynthetic efficiency and cell membrane integrity, and suppress plant growth and development, ultimately resulting in significant yield reductions and quality deterioration [3, 4, 5]. Cotton is a vital economic crop worldwide, occupying a cornerstone position in global agricultural production and the textile industry [6, 7]. While cotton exhibits moderate salt tolerance and serves as a pioneer species for saline‐alkali land remediation [8, 9], it remains highly vulnerable to salt stress, particularly during the seedling stage. This stage‐specific susceptibility stems from active cell proliferation and rapid vegetative growth, which render seedlings extremely prone to salinity‐induced physiological damage [10, 11]. Therefore, screening elite salt‐tolerant cotton germplasm during the seedling stage and elucidating the genetic mechanisms underlying salt tolerance can greatly contribute to the breeding of salt‐tolerant cotton varieties, improve the saline‐alkali soil utilization, and ensure the sustainability of cotton production.

Unraveling the genetic mechanisms of salt tolerance and identifying functional genes via genetic mapping represents a powerful and proven strategy for the molecular breeding of salt‐tolerant cotton. Genome‐wide association studies (GWAS) based on populations with rich genetic diversity have been extensively deployed to dissect the genetic basis of cotton salt tolerance, and a wealth of QTLs, SNPs, and candidate genes have been uncovered across diverse genetic backgrounds and growth stages [12, 13, 14, 15, 16]. However, traditional manual phenotyping methods are low‐throughput, costly, and prone to subjective bias, which greatly limit the efficiency and accuracy of genetic dissection for salt tolerance. Notably, the seedling stage is the most vulnerable period for cotton under salt stress [17, 18, 19]. Young seedlings undergo rapid growth and dynamic physiological fluctuations, and these subtle and dynamic changes are difficult to capture accurately by manual measurements. This highlights an urgent need for objective, high‐throughput phenotyping technologies to acquire precise and dynamic phenotypic data for salt tolerance research in cotton.

High‐throughput non‐destructive plant phenotyping technologies have undergone remarkable advancements in recent years, among which hyperspectral imaging (HSI) has established itself as a particularly transformative tool [20, 21]. HSI can capture continuous spectral information across visible to near‐infrared bands, covering the characteristic spectral signatures of key physiological and biochemical indicators in plants, such as pigments, water content, and metabolites. This technology enables comprehensive and non‐destructive characterization of the overall and internal physiological status of plants [22]. Under abiotic stress, plants exhibit subtle changes in spectral reflectance that are imperceptible to the human eye and undetectable by manual measurements. HSI can precisely capture these early and subtle stress‐responsive spectral signals, revealing the dynamic physiological responses of cotton under salt stress across cellular, tissue, and organismal scales, thus providing rich multi‐dimensional biological information for dissecting the genetic basis of complex agronomic traits [23, 24, 25]. Currently, HSI has emerged as a transformative approach for analyzing complex quantitative traits under abiotic stress. It has been successfully applied in major crops, including drought response in wheat [26], salinity tolerance in rice [27], and salt stress acclimation in cotton [28]. Integrating HSI with GWAS is conducive to breaking through the long‐standing “phenotyping bottleneck” and achieving high‐throughput and precise gene identification. Most existing studies treat hyperspectral phenotyping, gene identification, and functional verification as independent steps, lacking a standardized, coherent framework that directly connects non‐destructive phenotypes to functional genes. The combination of HSI‐derived data and GWAS has not been utilized to elucidate the genetic basis of salt tolerance in cotton.

In this study, we innovatively developed a framework that integrates phenomics, genomics, and functional validation to elucidate the genetic basis of salt tolerance. Time‐series hyperspectral imaging was employed to extract large‐scale *i*‐traits, enabling non‐invasive capture of the dynamic internal physiological responses of cotton seedlings to salt stress. A three‐tiered hierarchical screening strategy was then conducted to obtain core salt‐responsive *i*‐traits with biological relevance, genetic stability, and low redundancy suitable for genetic analysis. Using the screened core *i*‐traits as phenotypic inputs for genome‐wide association studies (GWAS), we successfully identified a key salt tolerance regulator and further validated its biological function and regulatory mechanism. Collectively, this study established an innovative framework from non‐destructive phenotyping to functional gene characterization, which enhances the efficiency and resolution of genetic dissection for complex stress traits and provides a generalizable research paradigm for similar studies in other crop species.

## 2 Materials and Methods

### 2.1 Plant Materials, Salt Treatment, and HSI Data Acquisition

419 accessions from a natural upland cotton population [29] were evaluated under control (CK) and salt treatment (ST), each with three biological replicates (Figure 1A, Table S1). After healthy seeds of each accession had been carefully selected, they were sown in plastic pots, each fitted with drainage holes to prevent waterlogging. Pots were arranged in a completely randomized design, and pot positions were rotated weekly to minimize positional effects. All phenotypic assays were performed in a precision climate‐controlled growth chamber with programmable temperature and humidity regulation. To better mimic field conditions, salt stress was imposed immediately after sowing by applying 200 mM NaCl solution, while control plants were irrigated with an equal volume of fresh water. The temperature was maintained at 30°C with a 16 h light/8 h dark photoperiod, and supplemental lighting was provided by broad‐spectrum LED lighting. After sowing, time‐series HSI data were acquired using a self‐developed high‐throughput HSI phenotyping platform on days 4 (emergence), 8, 12, and 16 post‐sowing (denoted as time 1–4). On time 4, physiological traits were measured concurrently for both the CK and ST groups, including plant height (PH), leaf chlorophyll content (LCC), fresh weight (FW), and dry weight (DW). PH was defined as the vertical distance from the growth substrate surface to the apical meristem. LCC was quantified spectrophotometrically following chlorophyll extraction from fresh leaf tissue using 95% ethanol. FW was determined by weighing individual seedlings on an analytical balance (accuracy: ± 0.001 g). Subsequently, each plant was placed in a pre‐labeled paper envelope, blanched in a 95°C oven for 30 min, followed by drying at 75°C to a constant weight. This drying process typically lasted 48–72 h, with consecutive weighings showing a mass difference of ≤ 0.002 g. Following drying, samples were transferred to a desiccator for cooling to room temperature and immediately re‐weighed on the same analytical balance to obtain DW.

**FIGURE 1:**
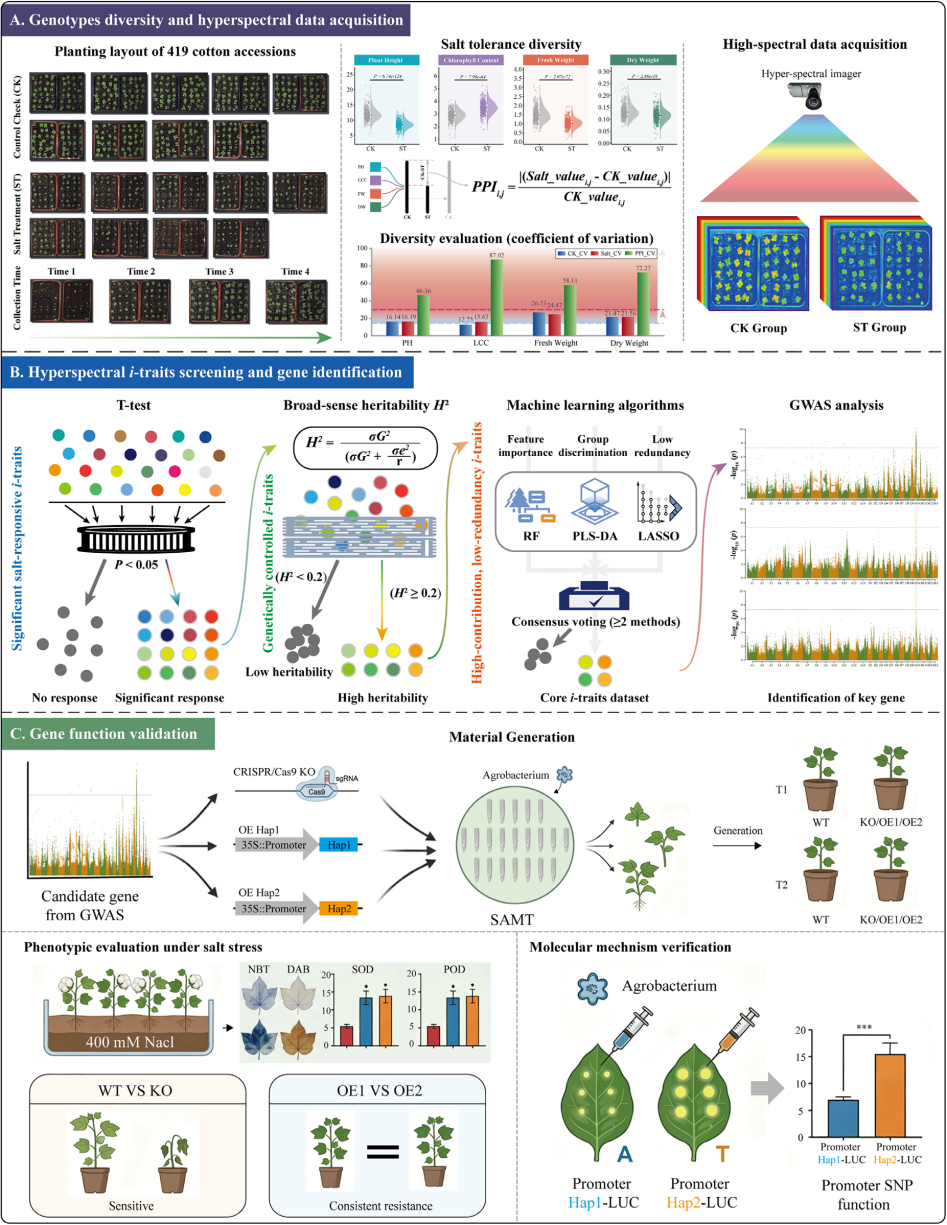
Overview of the study workflow. (A) Salt tolerance diversity of cotton accessions and hyperspectral data acquisition. The planting layout of 419 cotton accessions under control check (CK) and salt treatment (ST) across four time points is shown, alongside assessments of phenotypic diversity and plasticity index (PPI) to characterize salt tolerance variation. Hyperspectral imaging was performed to capture optical responses of plants under both treatments. (B) Hierarchically progressive *i*‐traits screening pipeline and gene identification. The pipeline comprises three sequential tiers: (1) t‐test to identify salt‐responsive *i*‐traits (*p* < 0.05); (2) broad‐sense heritability (H^2^ ≥ 0.2) estimation to retain genetically controlled *i*‐traits; (3) integration of three machine learning algorithms (Random Forest, PLS‐DA, and LASSO) via consensus voting to select high‐contribution, low‐redundancy core *i*‐traits. These core traits were then used as inputs for GWAS to identify key salt tolerance genes. (C) Functional validation of candidate genes via cotton genetic transformation. The workflow includes vector construction, shoot apical meristem transformation (SAMT) using ultrasound treatment, adventitious shoot induction, and regeneration of transgenic plantlets for salt tolerance verification.

### 2.2 Assessment of Salt Tolerance Diversity of Cotton Population Accessions

To assess salt tolerance diversity within the cotton population, we performed statistical analyses on key physiological indicators (PH, LCC, FW, and DW), which were measured under both CK and ST conditions (Figure 1A). These analyses enabled a clear visualization of phenotypic differences across treatments. Salt tolerance diversity is manifested not only in the absolute differences in physiological traits between CK and ST groups but also in “phenotypic plasticity under salt stress”, defined as the magnitude of change in each trait between the salt‐stressed and control conditions. Accordingly, we calculated the phenotypic plasticity index (PPI) for each of the four measured indicators [30, 31].

(1) $${\mathrm{PPI}}_{i,j}=\frac{\left|\left(\mathrm{Salt}\_{\mathrm{value}}_{i,j}-\mathrm{CK}\_{\mathrm{value}}_{i,j}\right)\right|}{\mathrm{CK}\_{\mathrm{value}}_{i,j}}$$

Where *i* denotes the accession ID, and *j* represents the physiological phenotypic trait.

To further validate population‐level phenotypic diversity, we computed the mean values of the PPI across the four physiological traits to derive the comprehensive phenotypic plasticity index (CPPI) [32]. This index was subsequently integrated with the coefficient of variation (CV) for comparative analysis. The CPPI is calculated as follows:

(2) $$\mathrm{CPPI}=\frac{\sum_{j=1}^{n}{\mathrm{PPI}}_{j}}{n}$$

Where *j* represents the physiological phenotypic trait, and *n* is the number of physiological phenotypic traits.

Principal coordinate analysis (PCoA) provides a low‐dimensional visual representation of the similarities and dissimilarities among samples [33, 34]. Therefore, PCoA of the PPI values derived from the four physiological characteristics was utilized to reveal the three distinct response clusters among the accession materials: high, medium, and low response categories.

### 2.3 Hyperspectral Data Acquisition

The hyperspectral reflectance data span the wavelength range of 400–1000 nm, encompassing both the visible (VIS) and near‐infrared (NIR) spectral regions. This range fully covers the primary spectral response region of plants and includes wavelengths strongly related to plant salt tolerance traits [35]. To ensure measurement consistency, data acquisition is conducted under controlled and uniform environmental lighting conditions. The illumination system employs halogen light sources powered by a stabilized DC power supply, maintaining light intensity stability with fluctuations of less than 0.1% per hour. Light is delivered symmetrically at a 45° incident angle from both sides, ensuring uniform spatial distribution across the sample surface (Figure 1A). The emitted spectrum fully overlaps the hyperspectral camera's operational wavelength band, exhibits no detectable flicker, and features low polarization. Before each data acquisition session, dark current correction and radiometric calibration are performed using a 99% reflectance diffuse white reference panel. The specific imaging process is as follows: (1) Open the HSI phenotypic platform, connect the computer, and launch the hyperspectral image acquisition software. (2) Place the cultivated cotton seedlings in the imaging box in sequence, and close the box door to capture the images. (3) Collect the RGB images and hyperspectral images of the seedlings, and save the data by date and group. (4) Use the hyperspectral image preprocessing software to analyze the collected data, which includes: seedling identification, binary segmentation and masking to isolate individual plants, hyperspectral *i*‐traits extraction, and *i*‐traits calculation processing.

### 2.4 Hyperspectral Salt‐Responsive *i*‐Traits Screening Pipeline

After acquiring the hyperspectral *i*‐traits of the CK and ST groups across the four time points, we developed a hierarchical screening pipeline for salt‐responsive *i*‐traits (Figure 1B): (1) Prioritizing biological significance. t‐tests were performed to identify *i*‐traits with significant differences between ST and CK groups across time‐series data. This study performed Student's t‐tests using a *p*‐value cutoff of 0.05 to determine statistical significance [36, 37]. Only hyperspectral *i*‐traits that showed statistically significant divergence between the ST and CK groups were retained to confirm their responsiveness to salt stress. This confirmed a biologically plausible association with salt stress while excluding features that arise stochastically and are unrelated to the salt stress response. (2) Evaluating genetic effectiveness. Given that GWAS requires traits to be under genetic control, the broad‐sense heritability (H^2^) was computed for all significantly different *i*‐traits [38, 39]. This step enabled the retention of genetically determined traits while mitigating confounding effects from environmental noise. The formula for H^2^ is as follows:

(3) $$H^{2}=\frac{\sigma G^{2}}{\left(\sigma G^{2}+\frac{\sigma e^{2}}{r}\right)}\;$$

where *σ*G² represents the genetic variance, *σ*e² represents the error variance. r represents the number of replicates, and if there is no repetition, r is set to 1. Only *i*‐traits with H^2^ ≥ 0.2 were retained.

(3) Integrating machine learning algorithms to improve screening efficiency. Statistical screening is a preliminary biological screening, while machine learning screening is a subsequent feature‐selection step for determining the importance of features. Both are indispensable. Machine learning can handle the collinearity of high‐dimensional data and capture nonlinear relationships. This study selected important hyperspectral features and removed redundant hyperspectral features by using feature importance analysis of machine learning. In this analytical module, three machine learning algorithms were integrated for robust feature selection: Random Forest (RF, PLS‐DA, and LASSO (Figure 1B). For the RF algorithm, feature importance was quantified using the Gini impurity. Higher importance values based on Gini impurity indicate a greater contribution of the *i*‐traits [40, 41]. For the PLS‐DA algorithm, a supervised discriminant model was constructed, and Variable Importance in Projection (VIP) scores were computed [42, 43]. The *i*‐traits with VIP > 1.2 were retained, indicating that their contribution to distinguishing the ST and CK groups exceeds the average explanatory power across all features. For the LASSO algorithm, leveraging L_1_ regularization, this method selects *i*‐traits associated with non‐zero regression coefficients, thereby promoting sparsity and interpretability [44]. Then, a consensus‐based fusion rule was adopted: only *i*‐traits selected by at least two of the three algorithms were retained for downstream analysis. Finally, through correlation analysis, redundant features with *r* > 0.8 were eliminated. This step not only retains the key information related to salt tolerance but also avoids false positive associations caused by multicollinearity in GWAS analysis.

Besides, to systematically reveal variations in response intensity across different accessions for the selected hyperspectral *i*‐traits, we constructed a hyperspectral *i*‐traits atlas, resulting in a clustering heatmap. To ensure inter‐trait comparability and eliminate scale effects, raw hyperspectral data were transformed into z‐scores using the formula:

(4) $$z\;=\frac{x-\mu }{\sigma }$$

Where x represents the original measurement value of certain *i*‐traits for this accession. *µ* and *σ* represent the average value and standard deviation of all accessions in the cotton population for the *i*‐traits, respectively.

This normalization procedure centers each *i*‐trait around a mean of zero with unit variance, allowing for the identification of relative deviations and synchronized response patterns across the entire population under salinity stress.

### 2.5 GWAS Analysis

A GWAS was performed for *i*‐traits to identify genetic associations between genomic polymorphisms and hyperspectral *i*‐trait phenotypes. SNPs were rigorously filtered using PLINK software [45], retaining 1,020,408 high‐quality SNPs, which served as the basis for subsequent GWAS analysis. GWAS analysis was conducted using the EMMAX algorithm. For visualization, GWAS results for the filtered hyperspectral *i*‐traits were plotted using the R package “CMplot”. A stringent genome‐wide significance threshold of ‐log_10_ (0.05/1,020,408) ≈ 7.30 was applied to control for multiple testing (Figure 1B).

### 2.6 Construction of Transgenic Materials

To investigate the functional differences between the two *GhST7* haplotypes and their potential regulatory roles, both overexpression and gene‐editing vectors were constructed in this study. Briefly, the coding sequences (CDS) of the two *GhST7* haplotypes were amplified from the cDNA of population materials. After sequence confirmation, the fragments were inserted into the WMV067 vector to generate the *GhST7* haplotype‐specific overexpression vectors. Subsequently, target sites within *GhST7* were predicted and screened using the CRISPR 2.0 online design tool. The sgRNA sequences were inserted into the SalI‐linearized WMV016 vector to construct the *GhST7* gene‐editing vector. The corresponding transgenic materials were generated using an ultrasound‐assisted shoot apical meristem transformation (SAMT) system, in which Agrobacterium‐mediated transformation was applied to the shoot apical meristem. These materials were used for subsequent functional validation and phenotypic analysis (Figure 1C).

### 2.7 RNA‐Seq Analysis

Cotton (*Gossypium hirsutum*) plants of the wild‐type (WT), *GhST7*‐overexpressing (*GhST7‐OE*), and *ghst7* lines were grown under normal conditions and subjected to salt treatment. Leaf samples were collected at 24 h after treatment, with three biological replicates for each genotype. Total RNA was extracted, and library construction and sequencing were performed. Sequencing was carried out on the Illumina NovaSeq 6000 platform, and approximately 6 Gb of raw data was obtained per sample. The raw reads were first assessed with FastQC for quality control and then trimmed with Trimmomatic to remove low‐quality bases and adapter sequences. The resulting clean reads were mapped to the reference genome TM‐1 (CRI) using HISAT2. Differential expression analysis was performed using DESeq2, and differentially expressed genes (DEGs) between pairwise comparisons (e.g., *GhST7‐OE* vs. WT and *ghst7* vs. WT) were identified with thresholds of FDR ≤ 0.05 and |log_2_(fold change)| ≥ 1.

### 2.8 Salt Stress Treatment of Transgenic Materials

To evaluate the physiological responses and salt tolerance of the transgenic lines, both control and transgenic plants were grown under identical conditions. After two weeks, the plants were treated with 400 mM NaCl for 72 h. Following treatment, phenotypic images were captured to document morphological responses to salt stress. In parallel, leaf samples were collected to measure superoxide dismutase (SOD), peroxidase (POD), and catalase (CAT) activities, as well as for nitroblue tetrazolium (NBT) and 3,3′‐diaminobenzidine (DAB) staining assays. These analyses were conducted to comprehensively assess reactive oxygen species accumulation and antioxidant defense capacity in the transgenic materials under salt stress.

### 2.9 Reactive Oxygen Species (ROS) Staining in Plant Tissues

ROS accumulation in plant tissues was detected using plant ROS detection kits based on either the DAB staining method or the NBT staining method. Leaves at the same developmental position were collected from different cotton materials at the seedling stage. Leaf discs of uniform size were then prepared using a leaf punch to ensure consistency in subsequent staining procedures. Leaf discs were submerged in DAB or NBT staining solutions and vacuum‐infiltrated for 30 min to promote solution infiltration, followed by overnight incubation in the dark at room temperature. After staining, the samples were retrieved with forceps and thoroughly washed 5 times with distilled water to remove any residual staining solution on the surface. The samples were then placed on filter paper to remove excess liquid, transferred into tissue decolorization solution, and incubated in a 70°C–80°C water bath for 40 min to eliminate background pigmentation in the leaves. After decolorization, the samples were rinsed again with distilled water 5 times, and the staining patterns were observed and photographed under a stereomicroscope to assess the accumulation level and spatial distribution of ROS in leaves from different plant materials.

### 2.10 Determination of CAT Activity

After salt treatment, CAT activity in the transgenic and control materials was determined using a Solarbio Catalase Activity Assay Kit. All experimental procedures were carried out following the manufacturer's instructions. For the reaction, 1 mL of CAT working solution was added to 35 µL of sample extract, and the initial absorbance at 240 nm was immediately recorded as A_1_. The absorbance was measured again after 1 min and recorded as A_2_. The change in absorbance was calculated as ΔA = A_1_ − A_2_.

(5) $$\mathrm{CAT}\left(U/g\right)=\frac{678\times \Delta A}{W}$$

where *W* represents the weight of the sample (g).

### 2.11 Determination of POD Activity

After salt treatment, POD activity in both transgenic and control materials was measured using a Solarbio POD Activity Assay Kit following the manufacturer's protocol. For this enzymatic assay, a 15 µL aliquot of enzyme extract was combined with a reaction mixture consisting of 270 µL distilled water, 520 µL reagent I, 130 µL reagent II, and 135 µL reagent III, followed by thorough mixing. The absorbance at 470 nm was recorded at 30 s (A_1_) and again at 1 min 30 s (A_2_). The change in absorbance was calculated as ΔA = A_2_ − A_1_.

(6) $$\mathrm{POD}\left(U/g\right)=\frac{7133\times \Delta A}{W}$$

where *W* represents the weight of the sample (g).

### 2.12 Determination of SOD Activity

After salt treatment, SOD activity in the transgenic and control materials was determined using a Solarbio SOD Activity Assay Kit based on the WST‐1 method. Briefly, 0.1 g of leaf tissue, ground thoroughly in liquid nitrogen, was weighed and homogenized in 1 mL of extraction buffer. The homogenate was then subjected to centrifugation at 8000 × g for 10 min at 4°C, and the resulting supernatant was harvested and used as the crude enzyme extract. Reaction mixtures for the sample tube, control tube, blank tube 1, and blank tube 2 were prepared according to the kit protocol and mixed thoroughly before analysis. The mixtures were then incubated in a water bath at 37°C for 30 min. After incubation, absorbance was measured at 450 nm and recorded as A_sample, A_control, A_blank1, and A_blank2, respectively. The absorbance differences were calculated as ΔA_sample = A_sample − A_control and ΔA_blank = A_blank1 − A_blank2. The inhibition percentage was calculated using the equation:

(7) $$\mathrm{SOD}\left(U/g\right)=\frac{1.11\;\times \;\mathrm{inhibition}\;\mathrm{percentage}}{\frac{1\;-\;\mathrm{inhibition}\;\mathrm{percentage}}{N}\times F}$$

where N represents the sample fresh weight and F represents the dilution factor.

### 2.13 Luciferase Reporter Gene Assay

The promoter fragments of two different *GhST7* haplotypes were cloned into the LUC‐containing 0800 vector to generate promoter‐driven luciferase reporter constructs. The recombinant plasmids were transiently co‐expressed in *Nicotiana benthamiana* leaves via *Agrobacterium tumefaciens*‐mediated infiltration. At 72 h post‐infiltration, luciferase activity was measured using a Tanon 5200 chemiluminescence imaging system.

## 3 Results

### 3.1 Salt Tolerance Diversity of the Cotton Population Based on Physiological Traits

To assess salt tolerance diversity within the natural population, this study measured four physiological traits (PH, LCC, FW, and DW) across all cotton accessions. Relative to the CK group, the ST group exhibited significant decreases in PH, FW, and DW, whereas LCC was markedly elevated under salt stress (Figure 2A). The corresponding PPI values were then calculated for each trait. The kernel density plots revealed that the PPI values for these four indicators spanned a wide range (0.00–2.50) and all exhibited pronounced multimodal distributions, indicating a high level of salt tolerance diversity within the population (Figure 2B). Subsequently, the coefficients of variation (CV) for the physiological trait measurements and PPI values were calculated separately to assess population diversity from two dimensions. The CV values for the four physiological traits in the CK and ST groups mostly exceeded 15%, indicating that the phenotypic diversity of the population accessions was rich under the same environmental conditions (Figure 2A). Under salt treatment conditions, the CV of PPI values captured dynamic response diversity, with values all exceeding 30%, demonstrating that the population exhibited a high degree of variation under salt stress conditions (Figure 2B, Table S2). These results confirm that the population fully meets the requirements for phenotypic diversity in subsequent hyperspectral *i*‐traits analysis and GWAS. To visually assess the gradation of salt tolerance diversity within the population, a PCoA analysis was performed based on PPI values. The results clearly revealed three clusters, corresponding to low, medium, and high‐performance categories, respectively (Figure 2C). Further calculation of CPPI values for these three clusters across the four physiological traits showed the same trend in expression levels, providing clear evidence of the rich salt tolerance diversity within the population (Figure 2D).

**FIGURE 2:**
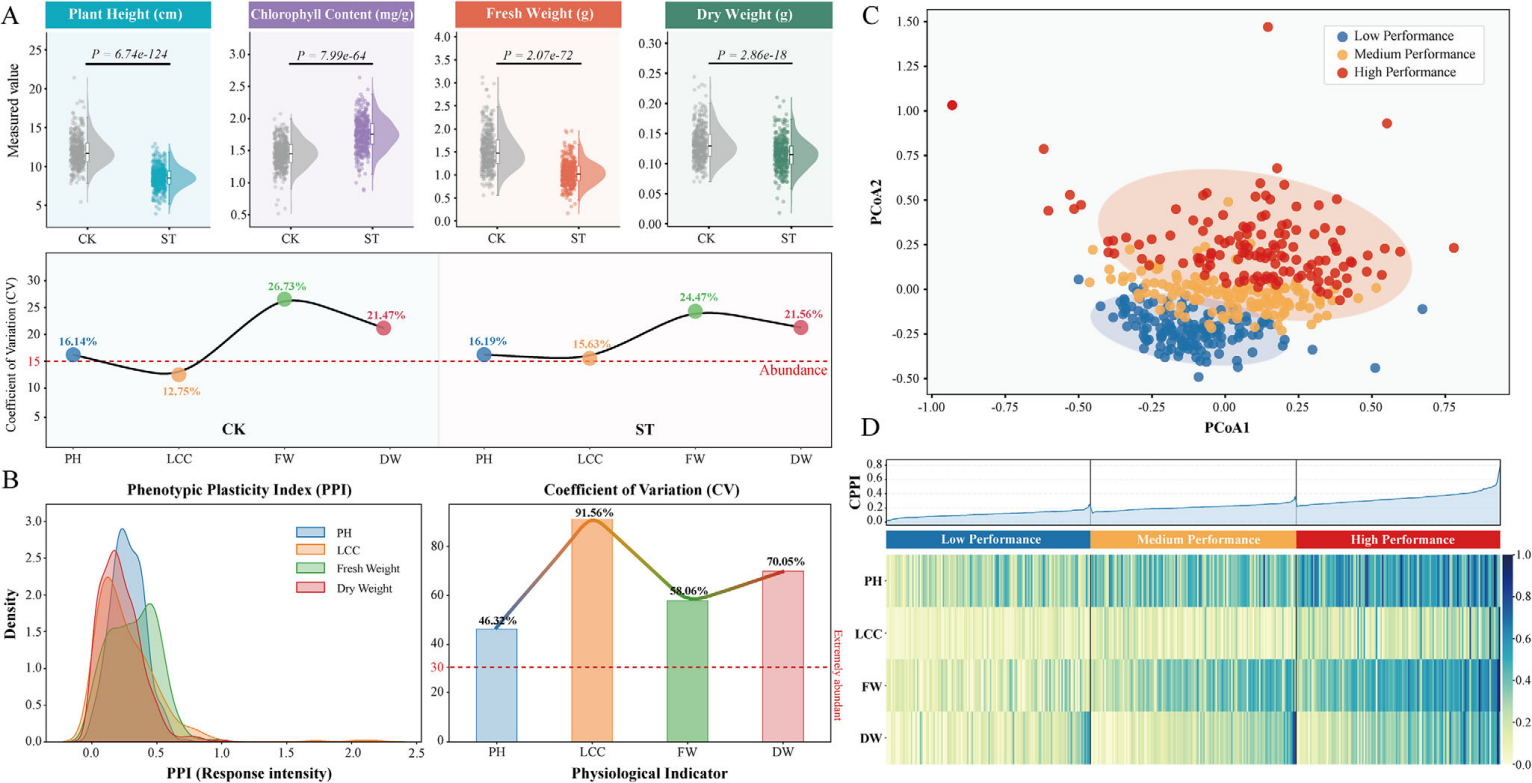
Phenotypic diversity and salt response plasticity of 419 cotton accessions under control and salt treatments. (A) The violin plot shows the distribution of four physiological traits (Plant Height, PH; Leaf Chlorophyll Content, LCC; Fresh Weight, FW; Dry Weight, DW) under control check (CK) and salt treatment (ST). Coefficient of Variation (CV) values for the four physiological traits in the CK and ST groups indicate that the phenotypic diversity of the population accessions was abundant. Significant phenotypic differences between treatments indicate population‐level salt responses. (B) Density distribution and CV of the Phenotypic Plasticity Index (PPI) for each trait. The kernel density plot illustrates the range of response intensity across accessions, while the bar plot shows the CV for PPI of each trait. (C) Panoramic heatmap of salt response plasticity across all accessions. Rows represent traits (PH, LCC, FW, DW), columns represent accessions sorted by CPPI, and color intensity indicates the magnitude of the PPI. (D) Principal Coordinate Analysis (PCoA) based on the physiological traits, showing the genetic and phenotypic diversity of the population. Accessions are colored by performance category, illustrating the separation of salt tolerance levels across the population.

### 3.2 Performance of the HSI Phenotyping Platform

To enable the acquisition of hyperspectral data during the seedling stage of cotton under salt stress, we have independently developed a high‐throughput HSI phenotyping platform (Figure 3A). It comprises an integrated hardware and software architecture designed to support the end‐to‐end workflow of hyperspectral data acquisition, preprocessing, and spectral feature extraction. The hardware system consists of a HSI enclosure, a black background board, a HSI hyperspectral imaging sensor, an RGB imaging sensor, aluminum rails, a hybrid illumination module, and a white calibration panel. The imaging enclosure features a fully enclosed structure (2.0 m × 1.5 m × 1.2 m), effectively shielding the imaging environment from ambient stray light and ensuring experimental reproducibility and measurement stability. The black background board is fabricated from matte, low‐reflectance material (< 1% reflectivity), thereby minimizing spurious background reflections that could otherwise contaminate target spectral signatures. The hyperspectral imaging sensor is a push‐broom type instrument covering a spectral range of 400–1000 nm, enabling accurate capture of fine‐scale spectral characteristics across the visible and near‐infrared regions (Figure 3B). The illumination module integrates halogen lamps and LED light sources: halogen lamps provide broad‐spectrum, spatially uniform irradiance across the visible to near‐infrared range for hyperspectral imaging, while LED lamps serve as supplementary sources to enhance illumination consistency and flexibility. The white calibration panel is constructed from a spectrally neutral, diffuse‐reflecting material with > 99% reflectance, facilitating radiometric calibration.

**FIGURE 3:**
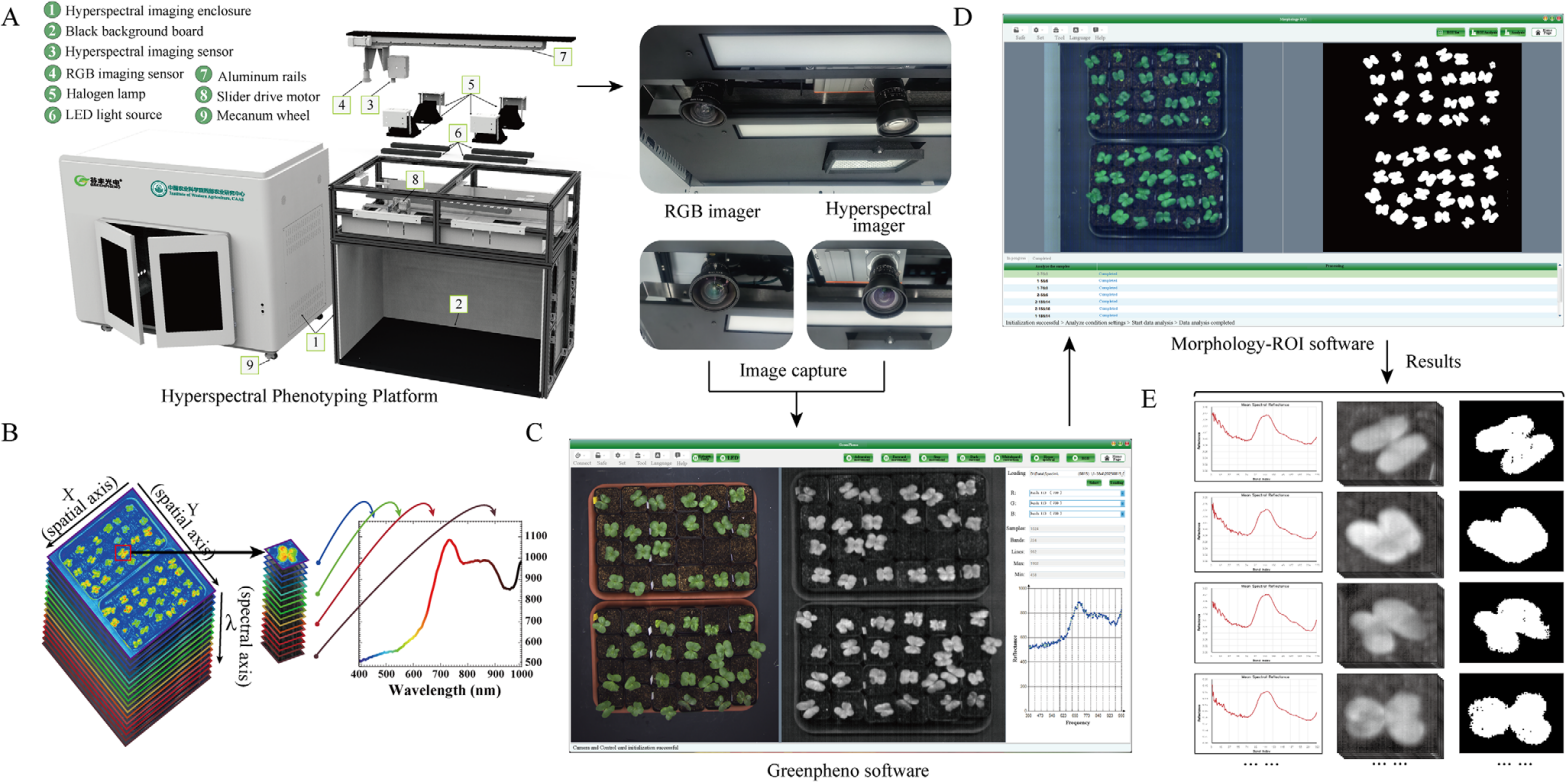
Hyperspectral imaging (HSI) phenotypic platform. (A) Hardware components of the plant hyperspectral imaging phenotyping platform. The integrated system includes an imaging enclosure with a low‐reflectivity black background board, a hyperspectral imaging sensor, an RGB imaging sensor, halogen lamps, LED light sources, aluminum rails, a slider drive motor, and a mobile wheel. The platform enables controlled, non‐destructive, high‐throughput acquisition of both visible and hyperspectral images of cotton seedlings under salt stress conditions. (B) Structure of a hyperspectral data cube. The cube illustrates the spatial (x, y) and spectral (λ) dimensions of hyperspectral data. Each pixel contains a continuous reflectance spectrum across the visible to near‐infrared range, capturing the unique spectral signatures of individual cotton plants. (C) Interface of the hyperspectral image acquisition software. The software supports real‐time detection of both RGB and hyperspectral images, enabling visual monitoring of plant growth and ensuring accurate data capture under standardized conditions. (D) Interface of the hyperspectral image preprocessing software. The software provides functions for plant segmentation and background removal, allowing automated extraction of the region‐of‐interest (ROI) for each seedling. (E) Example outputs of hyperspectral data analysis. The outputs include individual plant spectral profiles, grayscale images at specific wavelengths, and binary masks, which serve as the basis for subsequent extraction of thousands of hyperspectral *i*‐traits.

The software system contains two software programs to complete the entire process of data processing. The Greenpheno software is mainly used for the real‐time acquisition and preprocessing of hyperspectral and RGB images (Figure 3C). Those images provide accurate visual documentation of plant growth status across varieties and support region‐of‐interest (ROI) identification and segmentation in the corresponding hyperspectral data. The Morphology‐ROI software is responsible for the subsequent data analysis and feature extraction, enabling functions such as image segmentation, noise removal, and feature parameter calculation (Figure 3D). This software enables mask‐based segmentation for each accession and subsequent extraction of its hyperspectral information. The results include reflectance spectra, the segmented masks, and grayscale images corresponding to different spectral bands, as well as extracted *i*‐traits information (Figure 3E). The entire HSI platform forms a complete standardized process, starting from the automated collection of high‐throughput and multi‐temporal hyperspectral data, followed by image preprocessing, target area segmentation, spectral feature extraction and analysis processing, and finally, it can output thousands of phenotype‐related *i*‐traits for each genotype, providing a rich and precise data foundation for the analysis of genotype and phenotype correlations.

### 3.3 The *i*‐Traits Derived From HSI Data

Based on the HSI phenotyping platform's software system, automated spectral feature extraction from hyperspectral images was achieved, encompassing seedling identification, image binarization, ROI delineation, binary mask generation, and ROI‐wise band map segmentation (Figure 4A). Figure 4B displays the distribution of digital number (DN) values (representing the original image's radiometric quantization) of the leaf pixels across the spectral range, following mask‐based extraction. Leveraging this integrated hyperspectral platform, we achieved the extraction of hyperspectral *i*‐traits. After acquiring the binary HSI data stream for a single plant, 224 hyperspectral images were extracted from this stream. Subsequent image segmentation and trait quantification yielded a total of 1792 hyperspectral *i*‐traits, encompassing total reflectance, mean reflectance, and logarithm‐transformed traits, among others (Table S3). In this study, we collected multi‐temporal hyperspectral data of CK and ST groups from 419 cotton accessions grown, ultimately yielding a total of 14 336 HSI *i*‐traits under controlled indoor conditions to capture dynamic physiological response to salinity. These *i*‐traits were acquired via HSI and comprehensively capture plant spectral responses associated with salt tolerance. Therefore, they provide high‐resolution, quantitative spectral image information essential for downstream GWAS (Figure 4C). Subsequently, these *i*‐traits were subjected to a novel, purpose‐built hierarchical screening pipeline, which follows a progressive logic of “biological significance priority‐genetic effectiveness screening‐algorithm optimization for efficiency improvement”. Through this rigorous, multi‐tiered filtering process, we identified core *i*‐traits exhibiting strong and consistent associations with cotton salt tolerance.

**FIGURE 4:**
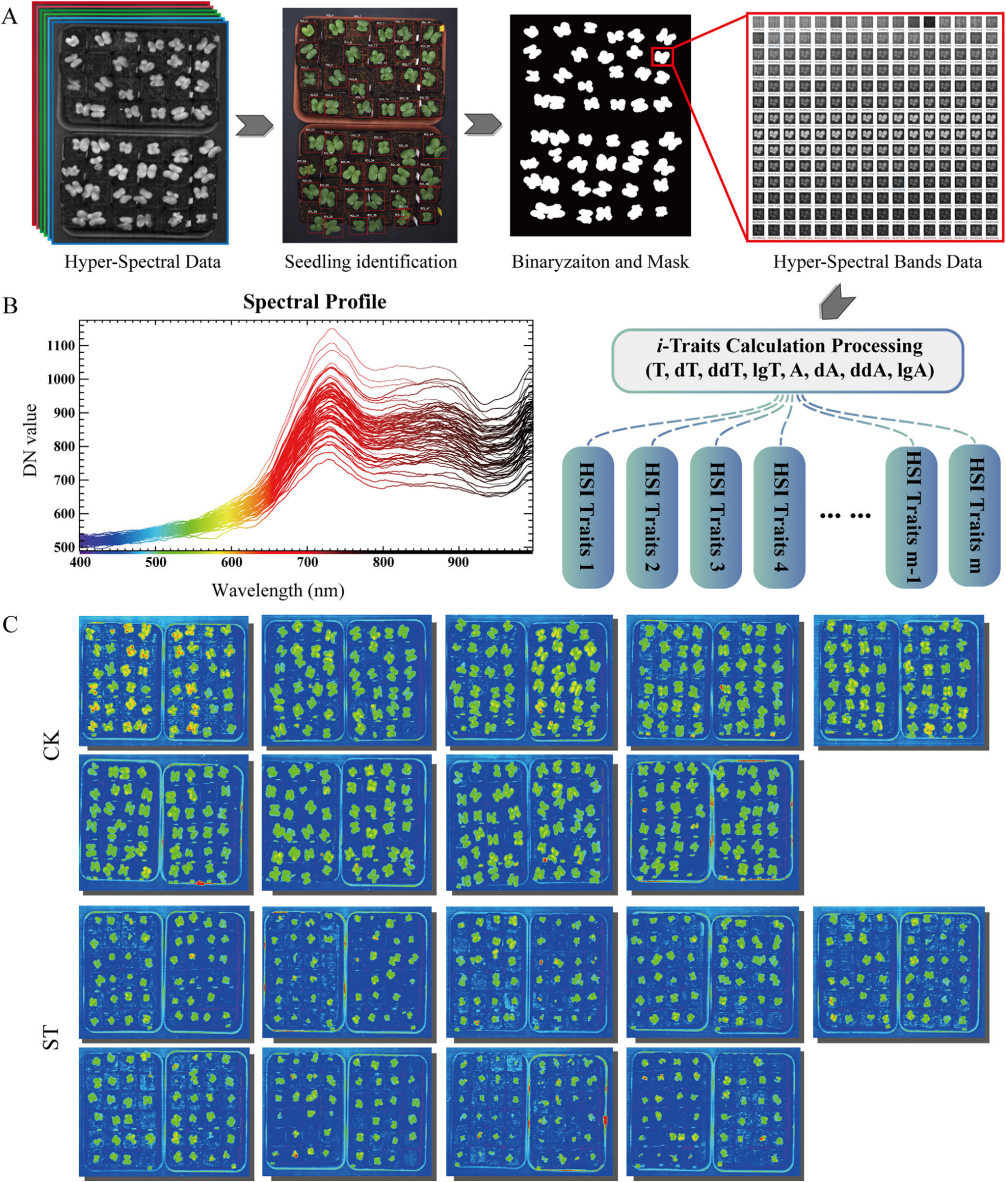
Workflow of hyperspectral image processing and *i*‐traits extraction. (A) Workflow illustrates the step‐by‐step pipeline for hyperspectral *i*‐traits extraction. Starting from raw hyperspectral data, the process includes seedling identification, binary segmentation, and masking to isolate plant pixels. (B) Spectral DN value profiles for each individual are extracted, and spectral traits are calculated using total reflectance (T) and other derived metrics to generate a comprehensive set of hyperspectral *i*‐traits. (C) One representative set of hyperspectral images of cotton seedlings under control (CK) and salt treatment (ST) conditions, captured at multiple time points, visually illustrates the physiological alterations induced by salinity stress.

### 3.4 Screening for Hyperspectral Salt‐Responsive *i*‐Traits Within Cotton Accessions

To identify robust *i*‐traits for salt stress response, this study performed a high‐throughput screening of 1792 hyperspectral *i*‐traits across four acquisition times using independent‐sample t‐tests. The 3D scatter plot (Figure 5A) provides a holistic view of the dynamic trajectory of salt‐responsive *i*‐traits. We observed that the discriminatory power of spectral traits was not static. Rather, it underwent a profound “expansion and intensification” as salt stress progressed. Specifically, while the early stage (Time 1, red clusters) exhibited localized sensitivity, the later stages (Time 2–4) demonstrated a widespread “spectral eruption” of significance across the entire index range, with −log_10_ (*p*‐value) magnitudes surging from initial significance to extreme levels exceeding 200. Further application of a multi‐segment broken‐axis visualization effectively partitioned the 1792 traits into distinct “responsive hotspots” (Figure 5B). A clear significance threshold (*p* < 0.05, red dashed line) successfully filtered out non‐responsive background noise (gray dots), isolating a core library of high‐sensitivity *i*‐traits (*n* = 800). Notably, certain spectral regions exhibited persistent hypersensitivity across all time points, suggesting these *i*‐traits capture physiological alterations caused by salt stress in cotton.

**FIGURE 5:**
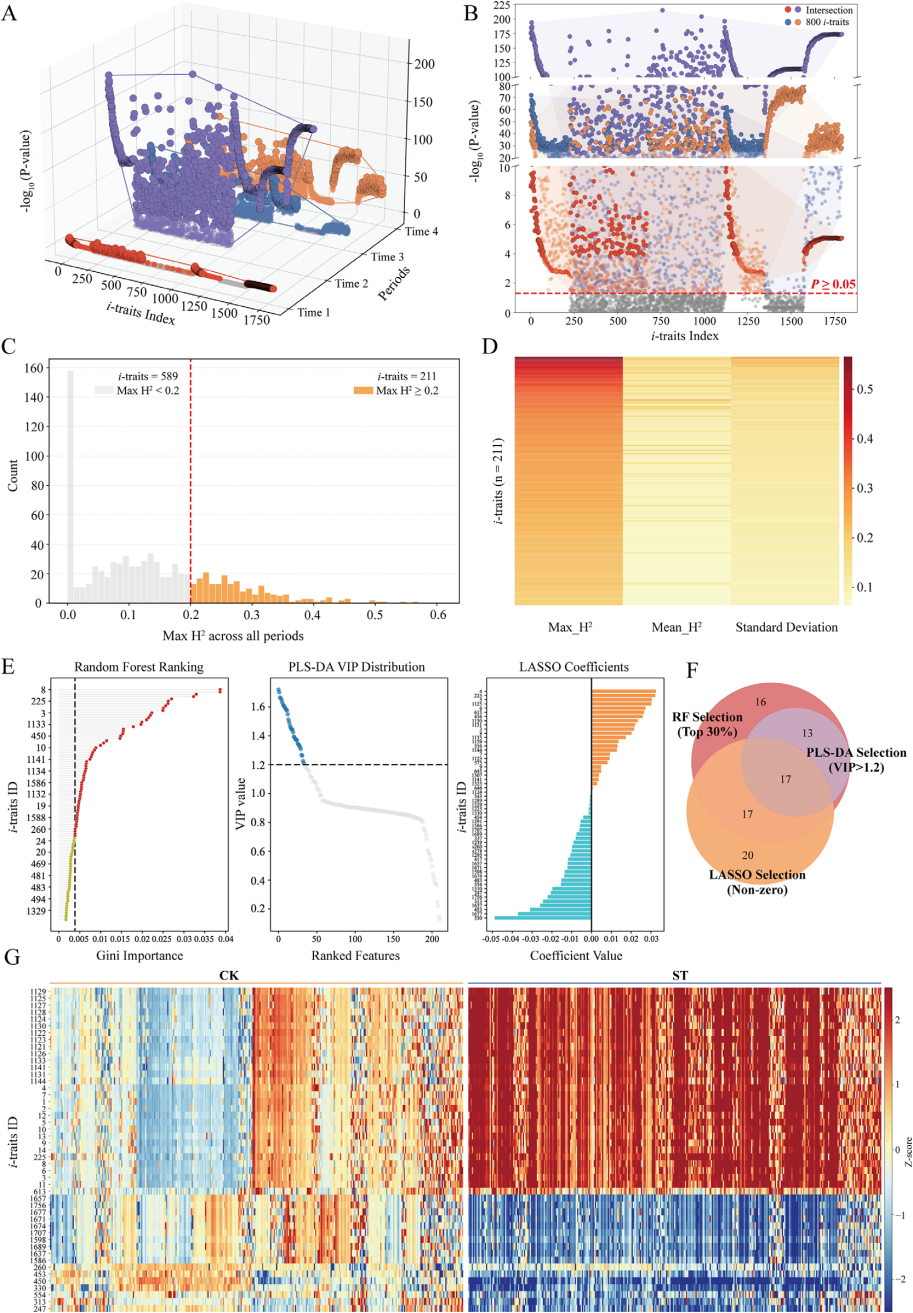
The hierarchically progressive hyperspectral *i*‐traits screening pipeline for salt tolerance in cotton. (A, B) T‐test analysis for salt‐responsive *i*‐traits. (A) A 3D scatter plot visualizes the significance (*p*‐value) of each *i*‐trait across different time periods. (B) A broken‐axis scatter plot further illustrates the distribution of significant responses at each time point, with different colors representing different sampling periods. (C, D) Broad‐sense heritability (H^2^) evaluation of *i*‐traits. (C) Histogram showing the distribution of maximum heritability (Max H^2^) across all periods, with a red dashed line indicating the threshold (H^2^ ≥ 0.2). (D) Heatmap presenting comprehensive heritability metrics (Max H^2^, Mean H^2^, and standard deviation) for each *i*‐trait. (E, F) Machine learning‐based core *i*‐trait selection. (E) Feature ranking by Random Forest (Gini importance), variable importance in projection (VIP) distribution from PLS‐DA, and non‐zero coefficient distribution from LASSO regression. (F) Venn diagram showing the consensus selection of core *i*‐traits by the three algorithms. (G) Hyperspectral *i*‐traits atlas under control (CK) and salt treatment (ST). The heatmaps display the standardized intensity (Z‐score) of each *i*‐trait across all accessions, revealing distinct hyperspectral response patterns under salt stress.

To verify the genetic control stability of hyperspectral *i*‐traits, we implemented a stringent H^2^ screening process. The distribution of maximum heritability (Max_H^2^) revealed a wide spectrum of genetic control, with values ranging from 0 to 0.6 (Figure 5C). In this study, a threshold of H^2^ ≥ 0.2 was applied [46], effectively bifurcating the feature space into noise‐prone environmental variables (gray bins) and genetically anchored phenotypic traits (orange bins). Notably, a robust subset of hyperspectral *i*‐traits exhibited persistent genetic stability, with 211 *i*‐traits surpassing the threshold, suggesting that these spectral signatures are strongly governed by the underlying genomic architecture rather than transient environmental fluctuations. Beyond static thresholding, we employed a novel multi‐dimensional evaluation matrix to characterize the heritability level of each *i*‐trait, integrating max_H^2^, mean heritability (mean_H^2^), and standard deviation (Figure 5D), providing a refined set of “genetically‐representative” traits for subsequent high‐resolution QTL mapping and genomic prediction.

To further improve screening efficiency and mitigate potential biases inherent in individual machine learning models, we implemented a synergistic ensemble selection method integrating RF, PLS‐DA, and LASSO regression (Figure 5E). RF importance ranking identified the high‐contribution *i*‐traits based on Gini importance. PLS‐DA quantified the discriminatory power of each trait, with a stringent threshold of VIP > 1.2 distinguishing key features that satisfied the filter criteria. LASSO regression performed feature shrinkage to identify *i*‐traits with non‐zero coefficients, where orange and cyan bars represented positive and negative regulatory contributions, respectively. Through the integration of the results from these independent algorithms, the 211 *i*‐traits were significantly reduced to 47 “core *i*‐traits” (voted by at least two algorithms) (Figure 5F). This multi‐dimensional voting mechanism ensures that the final core *i*‐traits are not mere artifacts of a specific algorithm but represent the most biologically and statistically significant hyperspectral features (Table S4).

The *i*‐traits atlas revealed a stark divergence in 47 *i*‐traits between the CK and ST. Within the CK group, different accessions displayed distinct spectral profiles. The left panel of Figure 5G reveals two clusters that exhibited significantly divergent high‐spectrum *i*‐trait responses. Overall, the CK group maintained a relatively homogeneous and stable phenotypic state. However, the introduction of salt stress triggered a comprehensive reconfiguration of *i*‐trait modules. Within the ST group, we observed distinct “salt‐responsive blocks”: a dominant cluster of *i*‐traits (upper right part) displayed a significant upward shift in intensity (Z‐score > 1.5) under stress. Conversely, another distinct cluster showed a synchronized decline of intensity in salt‐sensitive accessions. These high‐confidence features provided a streamlined and accurate input for subsequent GWAS analysis.

### 3.5 Integrated GWAS, Transcriptome, and VIGS Analysis Identifies GhST7 as a Salt Tolerance Regulator

To further dissect the genetic basis of salt tolerance, GWAS analysis was performed on 47 *i*‐traits at three time points. We set explicit screening criteria to filter GWAS‐suitable *i*‐traits from the 47 *i*‐traits: (1) The trait must produce genome‐wide significant association signals (−log_10_(P) > 7.30); (2) Significant SNP peaks detected at different time points must converge to the identical genomic interval on the same chromosome; (3) The corresponding spectral band has clear physiological interpretability. Only *i*‐trait 330, *i*‐trait 613, and *i*‐trait 1689 fully satisfied all three standards across all time‐series GWAS; hence, they were selected as representative indicators for candidate gene excavation. By integrating the association signals across time points and *i*‑traits, a genomic interval spanning 13.7–14.4 Mb on chromosome D10 was observed to be significantly related to salt stress tolerance (Figure 6A).

**FIGURE 6:**
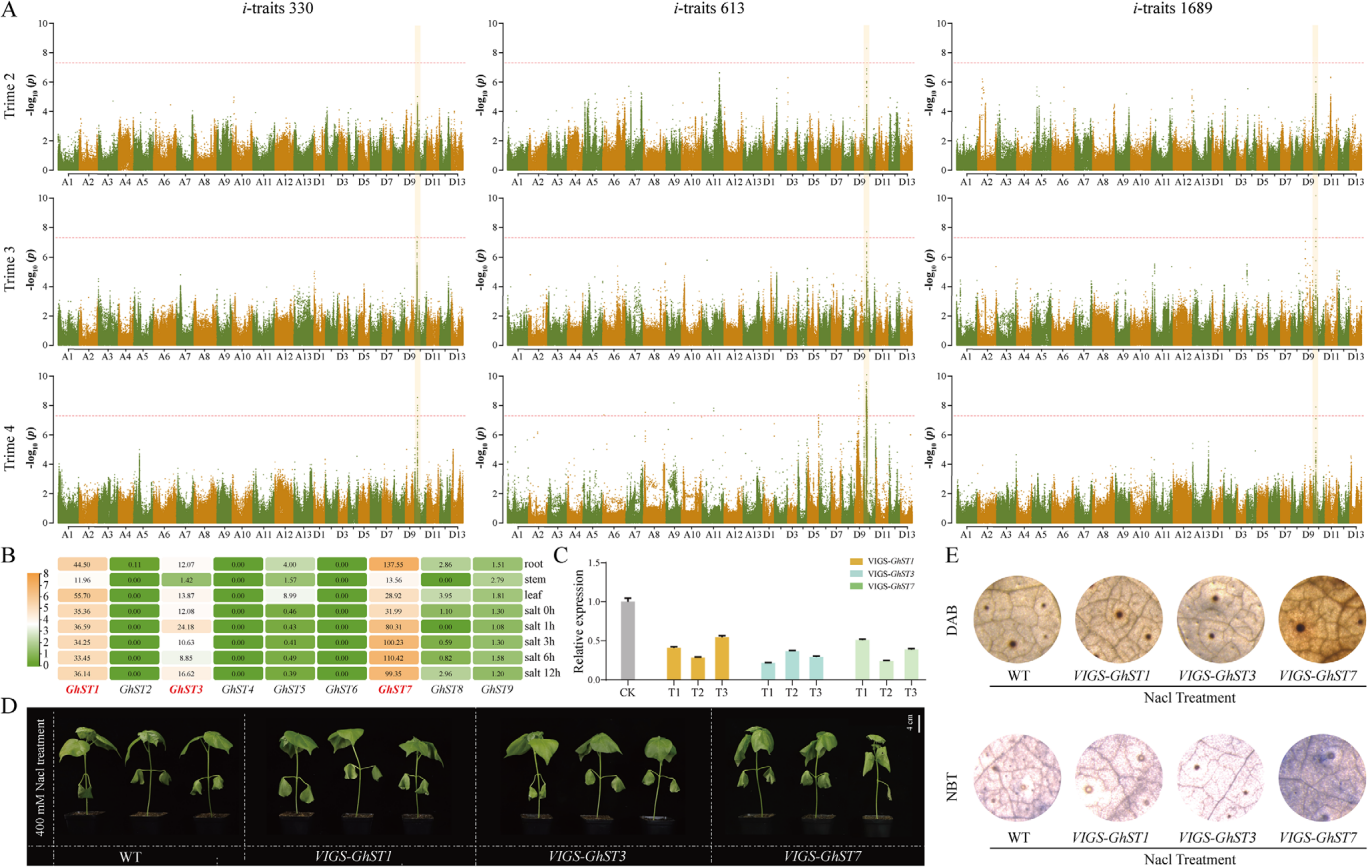
GWAS identifies *GhST7* as a candidate salt tolerance gene in cotton. (A) Manhattan plots of GWAS results for representative hyperspectral *i*‐traits across different time points. Each column corresponds to a representative core *i*‐trait (*i*‐trait 330, *i*‐trait 613, and *i*‐trait 1689), and each row represents a different time point (Time 2, Time 3, and Time 4). The y‐axis shows the −log_10_(*p*‐value) of each SNP association, and the red horizontal line indicates the genome‐wide significance threshold. The x‐axis displays the genomic positions of SNPs across the cotton genome. (B) Heatmap showing transcriptome expression profiles of genes located in the 13.7–14.4 Mb region of chromosome D10 in various tissues and under salt stress in Gossypium hirsutum. (C) Quantitative PCR analysis confirming the silencing efficiency of *GhST1*, *GhST3*, and *GhST7* expression by virus‑induced gene silencing (VIGS). (D) Phenotypes of wild‑type (WT) and *VIGS‑GhST1*, *VIGS‑GhST3*, and *VIGS‑GhST7* plants under 400 mM NaCl stress. (E) DAB and NBT staining of leaves from WT and *VIGS‑GhST1*, *VIGS‑GhST3*, and *VIGS‑GhST7* plants after 400 mM NaCl treatment, showing the accumulation of H_2_O_2_ and superoxide anions (O_2_^−^), respectively.

This interval contained nine candidate genes, designated *GhST1* through *GhST9* (Table S5). Using publicly available transcriptome data, we characterized their expression levels in roots, stems, and leaves, as well as their expression changes in response to salt stress. *GhST1* exhibited relatively high expression across all tissues, whereas *GhST3* and *GhST7* showed pronounced expression changes after salt treatment (Figure 6B). Based on these results, we performed a preliminary functional assessment of these three genes using a VIGS experiment. qRT‑PCR results confirmed that *GhST1*, *GhST3*, and *GhST7* expression levels were significantly suppressed in the respective silenced plants (Figure 6C). Following exposure to 400 mM NaCl, phenotypic observation revealed that *GhST7*‑silenced plants were more sensitive to salt stress (Figure 6D), which was further supported by DAB and NBT staining results (Figure 6E). Based on these findings, we selected *GhST7* as a potential regulator of salt stress tolerance for further functional characterization.

### 3.6 Natural Variation in the *GhST7* Promoter Regulates Gene Expression and Cotton Salt Tolerance

To functionally characterize the role of *GhST7* in cotton salt tolerance, two single‐guide RNAs (sgRNAs) targeting the first exon of *GhST7* were designed. Via the CRISPR/Cas9 genome editing system, three independent *ghst7* mutant lines with distinct editing profiles were successfully generated in the cotton accession ZM49. Sanger sequencing confirmed that all three mutants harbored insertions/deletions (indels) at the sgRNA1 target locus, which introduced frameshift mutations in the *GhST7* coding sequence (Figure 7A).

**FIGURE 7:**
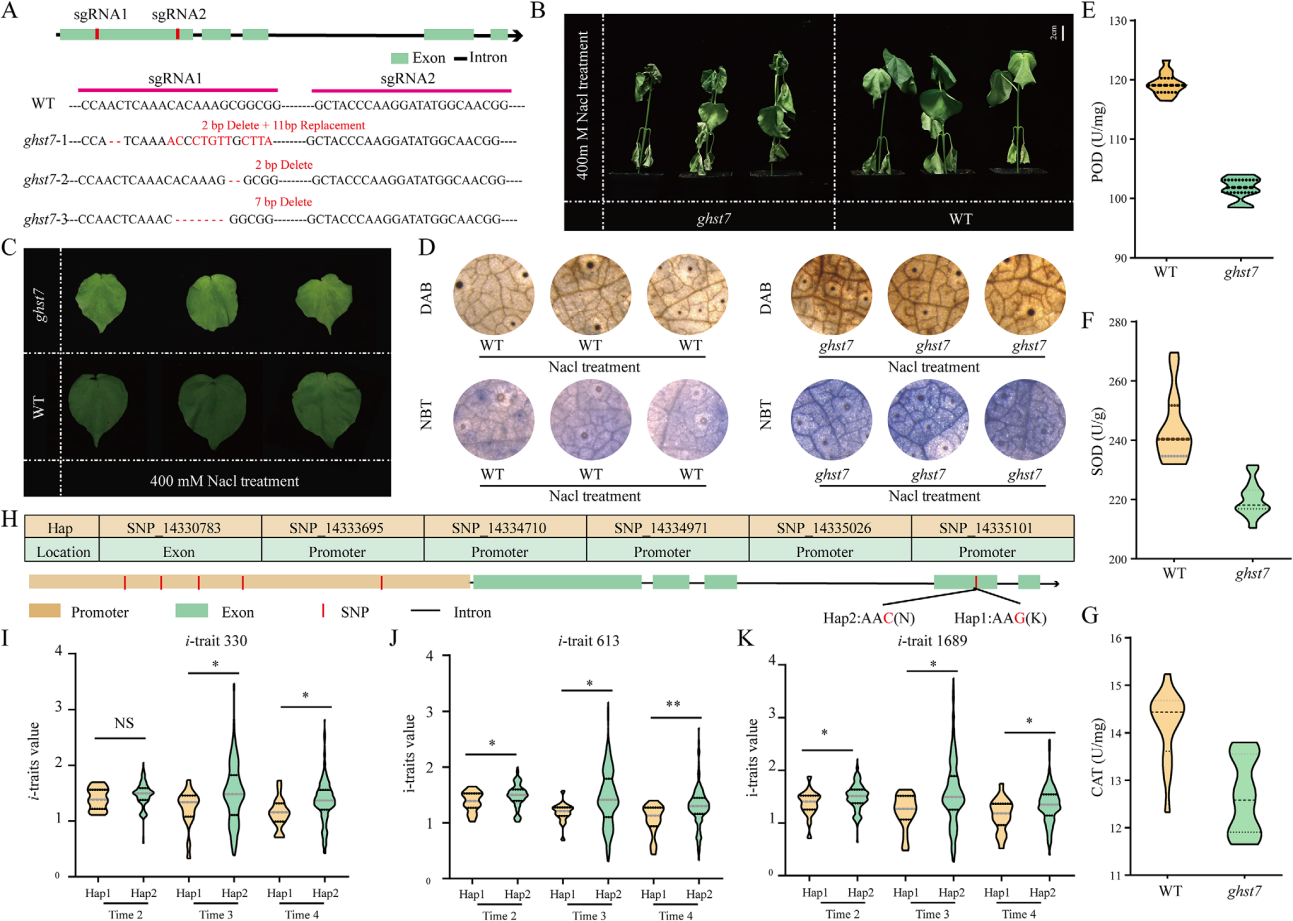
*GhST7* positively regulates salt stress tolerance in cotton. (A) CRISPR/Cas9 target sequence and editing patterns of the *GhST7* gene in the edited cotton lines. (B) Phenotypes of wild‑type (WT) and ghst7 mutant plants under 400 mM NaCl stress. (C) Leaf phenotypes of WT and ghst7 mutant plants after 400 mM NaCl treatment. (D) DAB and NBT staining of leaves from WT and ghst7 mutant plants following 400 mM NaCl stress, indicating H_2_O_2_ and O_2_^−^ accumulation, respectively. (E–G) Activity of superoxide dismutase (SOD) (E), peroxidase (POD) (F), and catalase (CAT) (G) in leaves of WT and ghst7 mutant plants under 400 mM NaCl stress. (H) SNP variation and haplotype analysis of the coding and promoter region of the *GhST7* gene. (I–K) Violin plots showing the distribution and statistical differences of two haplotypes for the hyperspectral image‑derived *i*‑trait 330 (I), *i*‑trait 613 (J), and *i*‑trait 1689 (K) at three time points.

Phenotypic analysis under 400 mM NaCl stress showed that the *ghst7* mutants were more sensitive to salt stress than the WT (Figure 7B), as evidenced by significantly increased leaf wilting and chlorosis after salt treatment (Figure 7C). DAB and NBT staining further revealed greater accumulation of H_2_O_2_ and O_2_^−^ in the *ghst7* mutant leaves, respectively (Figure 7D). Consistently, under salt stress conditions, all three major antioxidant enzymes (SOD, POD, CAT) displayed significantly attenuated activities in the *ghst7* mutants compared with WT plants (Figure 7E–G). Taken together, these results establish that *GhST7* is essential for cotton salt tolerance, and its functional loss leads to severe salt stress sensitivity.

We next assessed the impact of SNPs identified by GWAS on *GhST7* function. One nonsynonymous SNP was detected in the coding region, and five SNPs were identified in the promoter region of *GhST7* (Figure 7H). These SNPs were in linkage disequilibrium and formed two major haplotypes. Plants carrying the two haplotypes exhibited significant differences in the hyperspectral *i*‑trait 330, *i*‑trait 613, and *i*‑trait 1689 at three time points, except for *i*‑trait 330 at the first time point (Figure 7I–K), suggesting that these SNPs influence *GhST7*‑mediated salt tolerance.

To further assess whether the coding‑region SNP affects *GhST7* function, we generated two overexpression lines carrying the two coding‑region haplotypes (*GhST7‐OE‑Hap1* and *GhST7‐OE‑Hap2*). qRT‑PCR results confirmed that *GhST7* expression was markedly upregulated in both overexpression lines (Figure 8A). Under 400 mM NaCl stress, both *GhST7‐OE‑Hap1* and *GhST7‐OE‑Hap2* plants exhibited enhanced salt tolerance compared with WT (Figure 8B), as shown by less pronounced leaf wilting and yellowing (Figure 8C). DAB and NBT staining revealed reduced H_2_O_2_ and O_2_^−^ accumulation in the overexpression lines (Figure 8D,E). Furthermore, SOD, POD, and CAT activities were significantly increased in both *GhST7‐OE‑Hap1* and *GhST7‐OE‑Hap2* plants relative to WT (Figure 8F–H). Collectively, no statistically significant differences were detected between *GhST7‐OE‑Hap1* and *GhST7‐OE‑Hap2* in phenotype, ROS accumulation, or antioxidant enzyme activities, suggesting that the coding‑region SNP does not functionally affect *GhST7*‑mediated salt tolerance.

**FIGURE 8:**
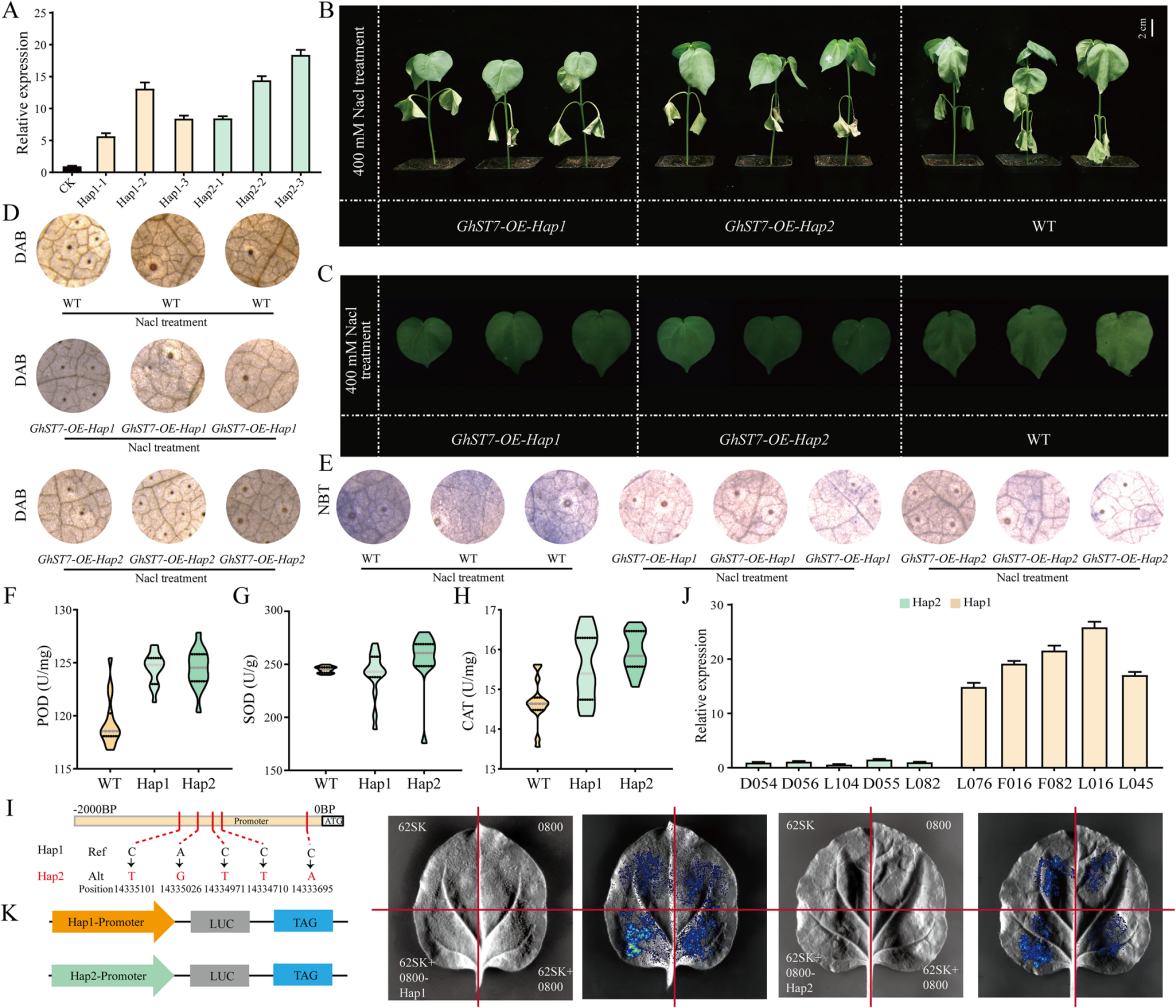
Promoter SNPs modulate *GhST7* expression and mediate *GhST7*‑regulated salt stress tolerance in cotton. (A) Expression analysis of *GhST7* in wild‑type (WT) and transgenic overexpression lines carrying two haplotypes (*GhST7‐OE‑Hap1* and *GhST7‐OE‑Hap2*). (B) Phenotypes of wild‑type (WT) and *GhST7‐OE‑Hap1/Hap2* plants under 400 mM NaCl stress. (C) Leaf phenotypes of WT and *GhST7‐OE‑Hap1/Hap2* plants after 400 mM NaCl treatment. (D‐E) DAB (D) and NBT (E) staining of leaves from WT and *GhST7‐OE‑Hap1/Hap2* plants following 400 mM NaCl stress, indicating H_2_O_2_ and O_2_^−^ accumulation, respectively. (F–H) Activity of superoxide dismutase (SOD) (F), peroxidase (POD) (G), and catalase (CAT) (H) in leaves of WT and *GhST7‐OE‑Hap1/Hap2* plants under 400 mM NaCl stress. (I) SNP variation and haplotype analysis of the *GhST7* promoter region. (J) qRT‑PCR analysis of *GhST7* expression levels in materials carrying different haplotypes. (K) Luciferase reporter assays detecting LUC expression driven by the *GhST7‐Hap1* and *GhST7‐Hap2* promoters.

Notably, the five SNPs located in the *GhST7* promoter region were in tight linkage disequilibrium, partitioning the natural population into two major haplotype groups (Hap1 and Hap2) (Figure 8I). qRT‐PCR results demonstrated that *GhST7* expression was markedly upregulated in Hap1 genotypes compared with Hap2 genotypes under salt stress (Figure 8J). To dissect the causal regulatory effect of these promoter variations on *GhST7* transcription, we constructed LUC reporter vectors driven by the full‐length promoter sequences of the two haplotypes, and found that the Hap1 promoter drove significantly higher LUC activity than the Hap2 promoter (Figure 8K). Together, these results demonstrate that natural variation in the *GhST7* promoter region regulates gene expression and thereby influences salt stress tolerance in cotton.

### 3.7 The Transcriptional Regulatory Role of *GhST7* in Plant Responses to Salt Stress

To investigate the transcriptional regulatory role of *GhST7* in plant responses to salt stress, we performed RNA‐seq analysis on WT, *GhST7‐OE*, and *ghst7* plants after salt treatment. Compared with WT, a large number of DEGs were identified in both *GhST7‐OE* and *ghst7* plants (Figure S1A,B). To further identify genes potentially directly mediated by *GhST7* under salt stress, we performed a joint analysis of the two transcriptomic datasets, focusing on genes up‐regulated in *GhST7‐OE* but down‐regulated in *ghst7*, as well as genes down‐regulated in *GhST7‐OE* but up‐regulated in *ghst7*. In total, 351 core genes were identified (Figure S1C). GO enrichment analysis showed that these core genes were significantly enriched in terms associated with salt stress response (Figure S1D). KEGG pathway enrichment analysis further demonstrated that the core genes were significantly enriched in signaling pathways, including the MAPK signaling pathway‐plant and the plant hormone signal transduction pathway (Figure S1E), with markedly altered expression of multiple related genes, such as PP2C and TMK (Figure S1F). Collectively, these results suggest that *GhST7* may participate in salt stress responses by modulating the expression of key genes in the MAPK signaling and plant hormone signal transduction pathways.

## 4 Discussion

### 4.1 Advantages of the HSI Phenotyping Platform for Salt Tolerance Monitoring in Cotton Seedlings

HSI has emerged as a well‐established non‐invasive tool for high‐throughput evaluation of plant abiotic stress responses, especially salinity [47]. Some studies have developed HSI‐based phenotyping platforms for assessing salinity tolerance across diverse crops, with substantial progress achieved in hardware construction and spectral data collection [48]. The HSI phenotyping platform established in this study provides a non‐destructive and standardized method for dissecting cotton salt tolerance in seedlings at the population scale. This integrated platform overcomes the inherent limitations of traditional manual measurements, including low efficiency, large human‐induced errors, destructive sampling, and poor reproducibility, thereby addressing the long‐standing phenotyping bottleneck in stress genetics research. From the perspective of hardware and software architecture, the hardware architecture ensures stable and high‐quality data acquisition. The enclosed dark environment eliminates interference from ambient light, while the low‐reflectivity background and illumination guarantee a high signal‐to‐noise ratio and reliable spectral capture. The push‐broom hyperspectral sensor enables fine‐grained recording of subtle spectral variations closely associated with leaf pigments and water status, which are critical for early and sensitive detection of salt stress responses invisible to the naked eye. For software, the existing platforms remain limited by insufficiently integrated or non‐standardized software pipelines, particularly for end‐to‐end batch processing and precise extraction of hyperspectral information under controlled stress conditions [49]. The supporting software developed in this study enables automated, high‐throughput, and standardized data processing, achieving accurate plant segmentation and high‐throughput extraction of large‐scale hyperspectral *i*‐traits. This end‐to‐end pipeline ensures consistent data processing across accessions, periods, and treatments, enhancing the comparability and repeatability of phenotypic data. To summarize, by enabling non‐destructive, high‐throughput, and high‐precision phenotyping for salt tolerance in cotton seedlings, this platform provides a robust technical foundation for capturing physiological responses, screening stable *i*‐traits, and improving the accuracy of subsequent GWAS.

### 4.2 The Scientificity, Rigor, and Efficiency of the Hierarchical Screening Pipeline for Salt‐Responsive *i*‐Traits

This study innovatively developed a hierarchical and progressive *i*‐traits screening pipeline to conduct a scientific and comprehensive screening of the obtained hyperspectral *i*‐traits. A three‐tiered screening strategy was implemented in the pipeline: (1) Statistical significance (t‐test): To isolate the “biologically meaningful salt‐responsive traits”, we identified *i*‐traits exhibiting statistically significant differences between the ST and CK groups. This step ensures biological relevance and eliminates invalid noise [50]; (2) Genetic determinism (H^2^): Focus on “heritable and suitable for GWAS traits”; retain only the traits that are controlled by genetics and have low environmental noise, enhancing GWAS power and interpretability [51]; (3) Feature optimality (ensemble machine learning): To select “highly predictive, non‐redundant core features”, we integrated RF, PLS‐DA, and LASSO algorithms in a consensus‐voting scheme. The result is more stable and reliable, while reducing redundancy, minimizing multicollinearity, and improving the efficiency of GWAS [52, 53, 54].

The hierarchical and progressive *i*‐traits screening pipeline, with each tier building upon the previous one and mutually verifying each other without being mutually exclusive, not only has biological significance but also possesses genetic reliability and statistical robustness. The innovation of this screening pipeline lies in its ability to condense complex, high‐dimensional spectral data into a decodable hierarchical matrix, allowing for the simultaneous evaluation of trait‐to‐trait correlations and variety‐specific resilience. By aligning the CK and Salt‐treated profiles in parallel, the pipeline effectively filters out noise and highlights “resilience fingerprints”, which can screen out accessions that maintain a balanced spectral profile despite high salinity. This pipeline enables the efficient, reliable, and scientific extraction of salt‐tolerance core *i*‐traits from a large number of hyperspectral features, providing a reference and replicable standardized process for future analysis of crop stress spectroscopic *i*‐traits.

### 4.3 The Advantages of Hyperspectral *i*‐Traits Over Traditional Phenotypes in Salt‐Tolerance GWAS

Compared with traditional phenotypic data, hyperspectral data offer substantial advantages. Traditional phenotypes (such as PH, FW, DW, etc.) are typically measured manually, a process that is inherently subjective, prone to measurement error, and often physically invasive to the plants [55]. In contrast, HSI‐derived *i*‐traits enable non‐contact, non‐destructive, and high‐throughput acquisition of optical data [20, 21, 22]. A single hyperspectral image captures thousands of *i*‐traits linked to both physiological and structural properties, including internal physiological changes imperceptible to the human eye. As demonstrated in Section 3.4, the generalized heritability (H^2^) of hundreds of hyperspectral *i*‐traits is consistently high and robust across environments, indicating that it is less affected by the environment and human interference and can provide a stronger, more reliable genetic signal. Moreover, due to the inherent optical properties of hyperspectral data, it can capture “hidden salt‐tolerance physiological characteristics” [56, 57]. Within the hyperspectral wavelength range, hyperspectral data contain rich, wavelength‐specific information highly responsive to key physiological characteristics such as plant chlorophyll, cell structure, water content, and osmotic potential [58, 59, 60]. These traits are inaccessible via traditional phenotyping yet are critically informative for salt tolerance assessment.

Based on the GWAS results, three optimal *i*‐traits with the most significant signals were identified as *i*‐trait 330, *i*‐trait 613, and *i*‐trait 1689. The spectral ranges where these three traits are located have important biological implications. (1) *i*‐trait 330 is derived from the red‐light band, which falls within the strong absorption band of leaf chlorophyll. This *i*‐trait mainly reflects chlorophyll content and the integrity of the photosynthetic apparatus under salt stress. Salt stress accelerates chlorophyll degradation and causes structural damage to the chloroplast apparatus in salt‐sensitive accessions. (2) *i*‐trait 613 is located in the near‐infrared band. Reflectance in this region is predominantly determined by internal leaf tissue structure, intercellular space scattering, and leaf water status. Under salt stress, sensitive accessions suffer osmotic injury, protoplast shrinkage, and collapsed mesophyll structure, which reduces near‐infrared scattering and reflectance. (3) *i*‐trait 1689 originates from the red‐edge band, highly sensitive to small fluctuations in chlorophyll and changes in cell turgor. It can capture early stress signals before visible damage appears on the leaf. Optically, salt‐induced chlorophyll loss and cell dehydration shift the red edge toward shorter wavelengths. Therefore, hyperspectral *i*‐traits facilitate the identification of salt‐tolerance‐associated genes that remain undetectable using traditional phenotypic metrics. Finally, compared to the single‐time‐point measurement of traditional phenotypes, the platform developed in this study enables rapid, convenient, and high‐throughput data collection across multiple time points. This temporal resolution provides a more comprehensive and dynamic characterization of cotton's internal salt‐tolerance mechanisms [61].

In summary, hyperspectral *i*‐traits, with their advantages of high throughput, non‐invasive nature, high stability, and high standardization, significantly improve the accuracy and dimensionality of salt tolerance phenotyping, serving as an ideal phenotypic source for GWAS of complex stress‐related traits.

### 4.4 Limitations and Future Work

The integrated analytical framework was developed and validated using a natural cotton population from under controlled indoor conditions. However, from the perspective of external validation, it still needs additional independent cotton populations or experimental batches for further validation. Although this study has fully verified the feasibility of the coherent phenomics‐genomics‐functional validation framework for cotton salt stress, the adaptability of this framework in different cotton populations under different genetic backgrounds still requires further exploration. Besides, the proposed phenotyping and screening pipeline is designed for indoor environments for high‐throughput germplasm screening. At present, the framework has been validated and applied in cotton. Although the screening logic based on statistical significance, heritability, and machine learning consensus is theoretically applicable to other crop species, its practical performance in other crops still requires independent experimental verification.

Further work will be carried out to address these limitations and refine the framework. We will introduce independent cotton populations with distinct genetic backgrounds and conduct multiple repeated planting batches under indoor conditions to systematically evaluate the stability and reproducibility of the hierarchical screening pipeline across different populations and experimental batches. In addition, we will extend this analytical framework to indoor seedling‐stage salt tolerance phenotyping of other major crops such as rice and wheat, to verify its cross‐species applicability and gradually establish a general paradigm for genetic dissection of complex stress tolerance traits based on hyperspectral *i*‐traits. Furthermore, integration with multi‐omics data will be performed to further deepen the biological interpretation of core *i*‐traits and improve the precision of gene mining for complex agronomic traits. Finally, for agronomic traits such as adult‐plant tolerance, reproductive performance, fiber yield and quality, multi‐stage field experiments and long‐term phenotypic observations are required to be conducted to advance crop improvement and field application.

## 5 Conclusion

This study employed 419 upland cotton accessions as experimental material and developed an integrated framework to dissect the genetic basis of salt tolerance. HSI technology was applied to capture *i*‐traits derived from salt‐tolerance hyperspectral images, and a hierarchical, progressive *i*‐trait screening process was conducted. The core salt‐responsive *i*‐traits identified through rigorous, multi‐tiered screening exhibited low redundancy and high explanatory power, thereby substantially enhancing the efficiency and gene mapping resolution of GWAS. This study establishes a coherent phenomics–genomics–functional validation framework, offering an efficient analytical paradigm and a valuable reference for precision genetic dissection of abiotic stress tolerance and molecular breeding in crops.

## Author Contributions

**Bowei Xu**: conceptualization, data curation, formal analysis, investigation, methodology, software, visualization, and Writing – original draft. **Le Liu**: formal analysis, methodology, visualization, and writing – original draft. **Mengli Yu**: data curation, formal analysis, visualization, investigation, and writing – original draft. **Yuling Guo**: data curation, investigation, and writing – original draft. **Junli Ye**: writing – review & editing, software. **Jun Zhang**: writing – review & editing, software. **Rumeng Zhao**: investigation and data curation. **Jiajie Yang**: investigation and data curation. **Wanneng Yang**: writing – review & editing. **Yameng Xu**: writing – review & editing. **Liqiang Fan**: Conceptualization, investigation, methodology, validation, and writing – review & editing. **Zuoren Yang**: funding acquisition and writing – review & editing.

## Conflicts of Interest

The authors declare no conflicts of interest.

## Supporting information




**Supporting File 1**: advs78504‐sup‐0001‐SuppMat.docx.

**Supporting File 2**: advs78504‐sup‐0002‐TableS1.xlsx.

**Supporting File 3**: advs78504‐sup‐0003‐TableS2.xlsx.

**Supporting File 4**: advs78504‐sup‐0004‐TableS3.xlsx.

**Supporting File 5**: advs78504‐sup‐0005‐TableS4.xlsx.

**Supporting File 6**: advs78504‐sup‐0006‐TableS5.xlsx.

## Data Availability

The data that support the findings of this study are available from the corresponding author upon reasonable request.
