# Supplementary material for: An Integrated Framework Coupling Hierarchical Hyperspectral i‐trait Screening, Gene Identification, and Functional Validation for Genetic Dissection of Salt Tolerance in Cotton
Source: Adv Sci (Weinh). 2026 Oct 26:e78504. Online ahead of print. doi: 10.1002/advs.78504 (PMC13710715; doi:10.1002/advs.78504)
Supplement: Supplementary file 1 [file ADVS-9999-e78504-s001.docx]

**Figure S1. RNA-seq analysis of *GhST7-OE* and *ghst7* lines after salt treatment.**

(A) Volcano plot showing the distribution of DEGs between *GhST7-OE* and WT after salt treatment. (B) Volcano plot showing the distribution of DEGs between *ghst7* and WT after salt treatment. (C) Venn diagram showing the number of DEGs common to the *GhST7-OE* versus WT and *ghst7* versus WT comparisons. (D) GO enrichment analysis of DEGs. From the inner to the outer ring, the circles represent the rich factor, the proportion of up-/down-regulated genes, the gene number, and the enriched GO term IDs, respectively. (E) Significantly enriched KEGG pathways of DEGs. (F) Schematic diagram showing expression changes of genes involved in the MAPK signaling pathway–plant and plant hormone signal transduction pathways.
